# RNA-seq reveals differentially expressed lncRNAs and circRNAs and their associated functional network in HTR-8/Svneo cells under hypoxic conditions

**DOI:** 10.1186/s12920-024-01933-4

**Published:** 2024-06-28

**Authors:** Jiaqing Zhou, YueHua Sheng, Zhezhan Chen, Huiqing Ding, Xiaojiao Zheng

**Affiliations:** 1 Obstetrics and Gynecology, Ningbo University, Ningbo, China; 2 Obstetrics and Gynecology, The First Affiliated Hospital of Ningbo University, Ningbo, China

**Keywords:** Hypoxia, HTR-8/Svneo, RNA-seq, PE, IUGR

## Abstract

Placental hypoxia is hazardous to maternal health as well as fetal growth and development. Preeclampsia and intrauterine growth restriction are common pregnancy problems, and one of the causes is placental hypoxia. Placental hypoxia is linked to a number of pregnancy illnessesv. To investigate their potential function in anoxic circumstances, we mimicked the anoxic environment of HTR-8/Svneo cells and performed lncRNA and circRNA studies on anoxic HTR-8/Svneo cells using high-throughput RNA sequencing. The miRNA target genes were predicted by integrating the aberrant expression of miRNAs in the placenta of preeclampsia and intrauterine growth restriction, and a ceRNA network map was developed to conduct a complete transcriptomic and bioinformatics investigation of circRNAs and lncRNAs. The signaling pathways in which the genes were primarily engaged were predicted using GO and KEGG analyses. To propose a novel explanation for trophoblastic organism failure caused by lncRNAs and circRNAs in an anoxic environment.

## Background

The placenta is a pregnancy-only organ that aids in the passage of nutrients and gases between the mother and fetus. The chorionic trophoblast must reconstruct the spiral artery for the placenta to form [1, 2].

Placental hypoxia is linked to a number of pregnancy illnesses, the majority of which occur in the middle and late stages of pregnancy [3]. Preeclampsia(PE) and intrauterine growth restriction(IUGR) are two maternal disorders caused by placental hypoxic stress. Preeclampsia is a complication of pregnancy with a global incidence of about 4–5% [4]. PE usually presents after 20 weeks of gestation, and the main clinical manifestations are new-onset hypertension and proteinuria. Many studies believed that the pathogenesis of preeclampsia is related to the placental dysfunction [5, 6]. Intrauterine growth restriction refers to a fetus that has failed to reach its biological growth potential because of placental dysfunction.It is a leading cause of infant morbidity and mortality. The most commonly observed placental disease in association with IUGR is maternal vascular malperfusion [7, 8].

Many investigations have demonstrated that placental hypoxia is caused by uterine artery recasting problems and insufficient penetration of extracellular villus trophoblast cells [9]. The stress response releases a number of placenta-derived substances into the blood, inducing immunological and vasculitic reactions as a result of the placenta’s ongoing hypoxia. Maternal hypertension, fetal hypoxia, and other clinical manifestations then ensue. Placental hypoxia plays a crucial role in the initiation and development of PE and IUGR [10, 11]. It was discovered that PE had higher levels of the hypoxia-related factor HIF1a expression [12]. Low levels of PLGF expression were found in the PE, and IUGR serum [13, 14]. Unfortunately, there is no clinical indicator that can accurately capture how much placental hypoxia is present.

A class of noncoding circRNAs known as circRNAs are primarily found in the cytoplasm. Recent research has demonstrated that circRNAs participate in pathophysiological processes through a variety of routes, the more prevalent of which is the ceRNA pathway, which can act as a molecular sponge for miRNAs and influence their function [15–17].

LncRNAs are a class of long, highly conserved noncoding RNAs that are mostly expressed in the nucleus and cytoplasm and have a length larger than 200 bp [18, 19]. A variety of mechanisms, including the ceRNA pathway and the direct control of protein production, are used by lncRNAs to function [15]. In HTR-8/Svneo cells, lncRNA controls invasion, proliferation, and apoptosis, according to previous studies [20]. It is unclear at this time how circRNAs and lncRNAs are expressed in placental hypoxia.

Our study aimed to understand the role of lncRNAs and circRNAs in placental development and pregnancy-related processes, and the HTR-8/Svneo cell line from human trophoblast cells is a suitable model. In addition, The HTR-8/Svneo cell line have been extensively characterized in previous studies, providing a rich foundation of existing knowledge about its behavior, gene expression patterns, and functional properties. This prior understanding can facilitate the interpretation and comparison of experimental results. They can provide valuable insights into cellular processes and molecular mechanisms.

High-throughput gene sequencing was used in this study to examine the diverse ways that lncRNAs, circRNAs, and mRNAs were expressed in anoxic HTR-8/SVneo cells. Although our methods and experiments are similar to those in some papers, previous studies have mainly focused on tumor tissue [21, 22]. We did not find similar studies in the HTR-8/SVneo cell line.The coexpression of these dysregulated RNAs and ceRNA networks was investigated to anticipate the function of lncRNAs and circRNAs.

## Methods

### Cell cultures

The human chorionic trophoblast cell line HTR-8/Svneo (purchased from Cell Bank of Chinese Academy of Sciences, China) was cultivated in media that also contained 1% and 10% penicillin‒streptomycin (RPMI-1640, Corning, Cellgro, USA) and fetal bovine serum (FBS, Pan-biotech, Germany ). They were cultivated in a typical, humidified tissue culture chamber (normoxic) at 37 °C, 21% O2, and 5% CO2. Cells were cultivated in an anoxic chamber that was humidified (made by Forma Scientific, Marietta, Ohio, USA) at 37 °C, 1% O2 and 5% CO2.

### RNA extraction

RNA was extracted from control and hypoxic HTR-8/SVneo cells using TRIzol reagent (Invitrogen, Carlsbad, CA, USA) 24 h after culture according to the manufacturer’s instructions. The concentration of total RNA was determined by a NanoDrop ND-1000 spectrophotometer (Thermo, Waltham, MA, USA).RNA Integrity and gDNA contamination test by denaturing agarose gel electrophoresis (concentration of agarose gel is 1%, and 0.5 µg/ml ethidium bromide to help it visualized).

### High-throughput sequencing

Based on high-throughput sequencing results, transcriptome sequencing and subsequent bioinformatics analysis were conducted by Kangcheng Biotechnology (Shanghai, China). The library was constructed with a KAPA Stranded RNA-Seq Library Prep Kit (Illumina) after total RNA samples were stranded by oligo dT enrichment (rRNA removal). In the process of library construction, double-stranded cDNA was synthesized using the dUTP method combined with subsequent high-fidelity PCR polymerase action, which made the resulting RNA sequencing library have chain specificity. The constructed library was evaluated by an Agilent 2100 Bioanalyzer and quantified by qPCR. The mixed libraries of different samples were sequenced using an Illumina NovaSeq 6000 sequencer.

### Sequencing analysis of lncRNAs, circRNAs, and mRNAs

Based on preliminary judgment and recognition, paired-end reads were obtained from an Illumina NovaSeq 6000 sequencer. The sequencing quality of the reads was evaluated by FastQC software, and cutadapt was used to remove the 3’ and 5’ joints. LncRNAs, circRNAs, and mRNAs were analyzed using high-quality pruned read segments by comparing them to the reference genome (GRCh37) using HISAT2 software.

### Statistical analysis

Statistical analyses were performed using R software (3.5.0). Fisher’s exact test was used to determine the statisticalsignificance for comparison of two groups. Pearson’s test was used for the correlation analysis. *P* < 0.05 was considered statistically significant.

### Differential expression of lncRNAs, circRNAs and mRNAs

FPKM calculations at the gene level and transcription level were performed using Ballgown, and differences in expression at the gene level and transcription level were calculated separately. We considered multiples ≥ 2.0 with *p* < 0.05 and FPKM values ≥ 0.1 in at least one sample from one group to indicate differentially expressed lncRNAs, circRNAs, and mRNAs.

### GO analysis and KEGG enrichment

GO and KEGG analyses were used to predict the biological functions of lncRNAs, circRNA target genes and mRNAs. GO analysis was used to identify and annotate differentially expressed genes in three categories: molecular function (MF), biological process (BP) and cell component (CC). KEGG enrichment analysis is an important way to analyze target genes and mRNAs involved in the differential expression of lncRNAs and circRNAs. The error detection rate (FDR) was used to correct p values. A p value < 0.05 was considered statistically significant enrichment.

### Construction of a ceRNA network

To further understand the potential interaction of differentially expressed lncRNAs, circRNAs and mRNAs, we constructed a ceRNA network. Differential lncRNA, circRNA, mRNA were obtained by sequencing. The common differentially expressed miRNAs in placentas of PE and IUGR were screened in the NCBI (National Center for Biotechnology Information (nih.gov)), and the interactions between different lncRNAs, circRNAs and miRNAs were predicted based on popular miRNA target gene prediction software. Subsequently, miRNA binding sites and target miRNAs were predicted using proprietary software based on TargetScanHuman 8.0 (https://www.targetscan.org/vert_80/, Whitehead Institute, Cambridge, MA, USA) and miRanda (http://mirtoolsgallery.tech/mirtoolsgallery/node/1055). Therefore, lncRNA-mRNA, circRNA-mRNA, and lncRNA-miRNA-circRNA-mRNA networks were constructed using Cytoscape (v3.7.2, National Institute of General Medical Sciences, Bethesda, MD, USA) software based on lncRNAs, miRNAs, circRNAs and mRNAs.

## Results

### Differential expression of circRNAs, lncRNAs and mRNAs in HTR-8/SVneo cells in an anoxic environment

**Fig. 1:**
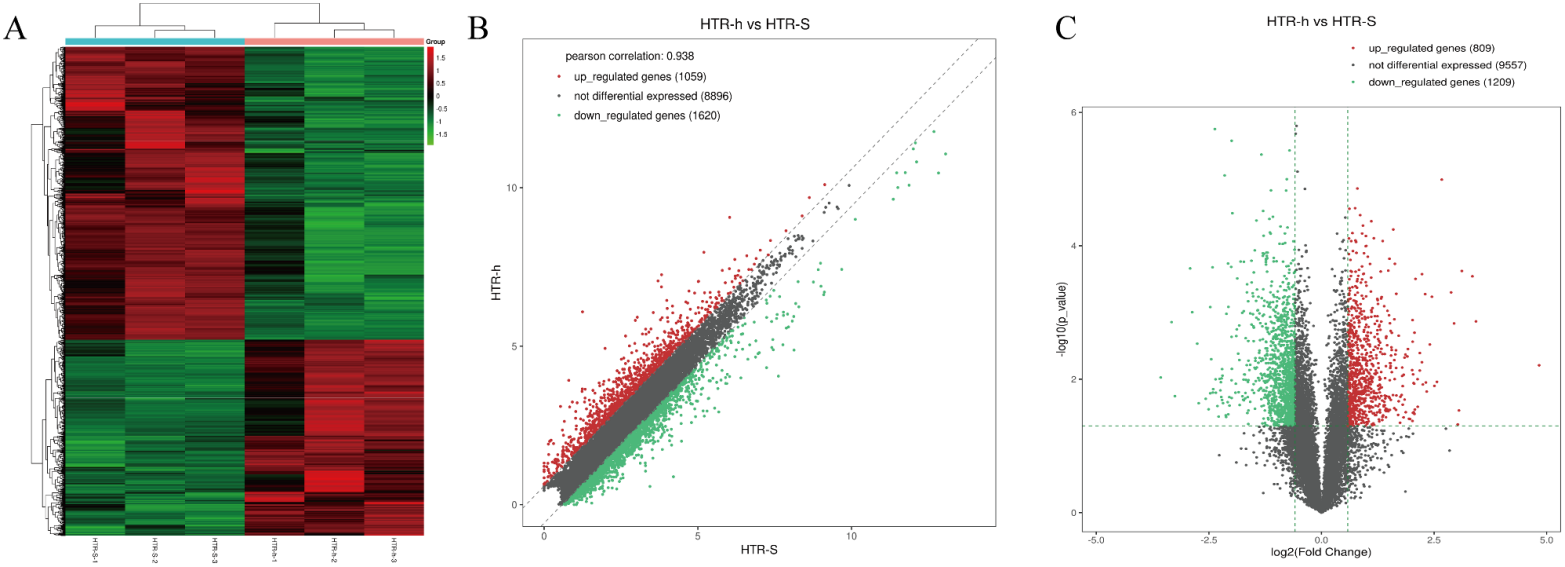
Identification of differentially expressed genes by RNA-seq analyses in HTR-8/SVneo cells. **A**. Clustered heatmap of the differentially expressed genes in three paired normoxic andhypoxic HTR-8/SVneo cells. Rows represent genes, while columns represent cell. **B**. The scatter plot shows the distributions of mRNAs in a more direct way. **C**. Volcano plot showing the significantly differentially expressed genes

**Fig. 2:**
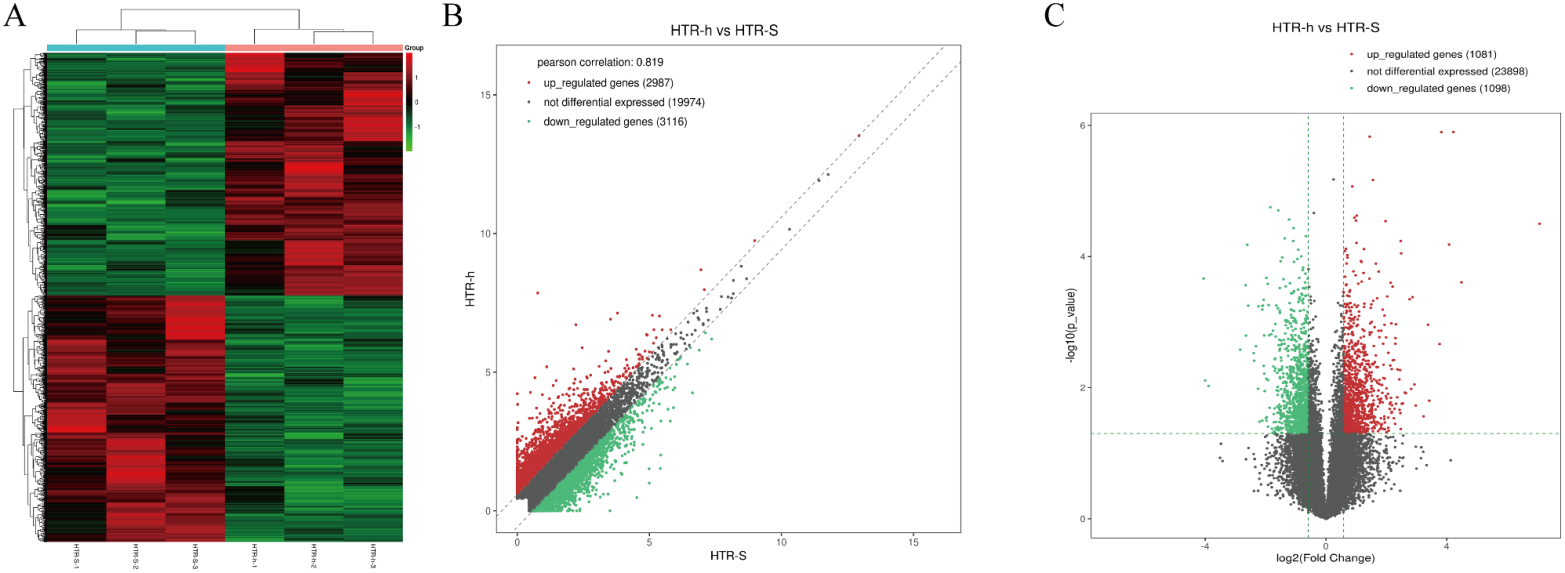
Identification of differentially expressed genes by RNA-seq analyses in HTR-8/SVneo cells. **A**. Clustered heatmap of the differentially expressed genes in three paired normoxic and hypoxic HTR-8/SVneo. Rows represent genes, while columns represent cell. **B**. The scatter plot shows the distributions of lncRNAs in a more direct way. **C**. Volcano plot showing the significantly differentially expressed genes

**Fig. 3:**
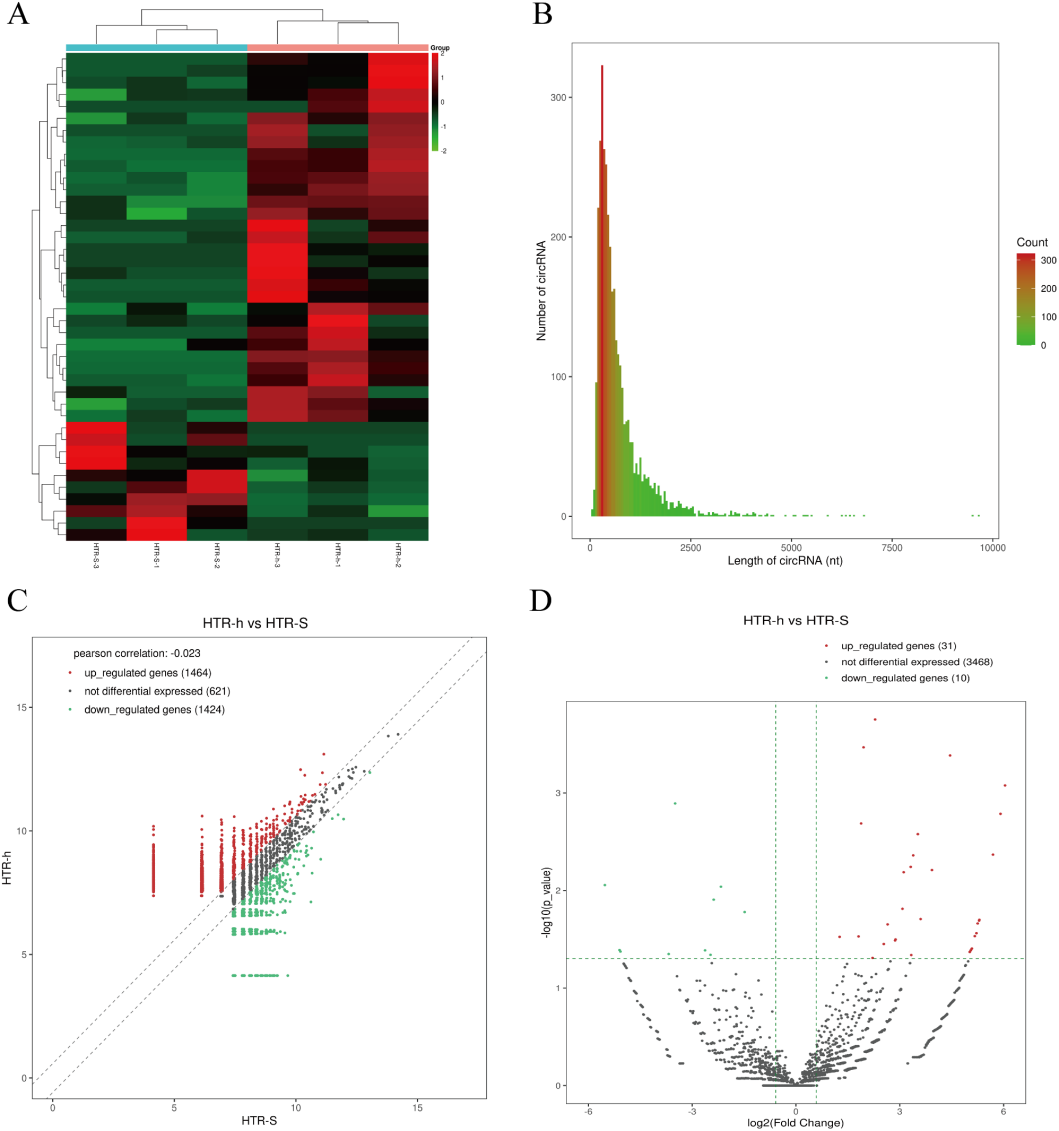
Identification of circular RNAs by RNA-seq analyses in HTR-8/SVneo cells. **A**. Clustered heatmap of the differentially expressed circRNAs in three paired normoxic and hypoxic HTR-8/SVneo cells. Rows represent circRNAs, while columns represent cell. **B**. The length distribution of exonic circRNAs. **C**. Scatter plot showing the distributions of circRNAs in a more direct way. **D**. Volcano plot showing the significantly differentially expressed circRNAs

RNA sequencing was used to find thousands of HTR-8/Svneo transcripts expressed in normoxic and anoxic environments. In all, 885 mRNAs were upregulated, and 1378 mRNAs were downregulated, resulting in the differential expression of 2263 mRNAs and 2179 lncRNAs (fold change ≥ 2 and *P* < 0.05) (Fig. 1). A total of 1081 lncRNAs were upregulated, while 1098 were downregulated (Fig. 2). The heatmap of circRNA differentially expressed genes revealed that the majority of them were shorter than 2000 nt. In total, the heatmap and scatter plot of the various circRNA expression patterns revealed 41 significant variations in circRNA. In the anaerobically treated HTR-8/Svneo cells, 31 genes were upregulated, and 10 genes were downregulated (Fig. 3).

### Functional analysis

**Fig. 4:**
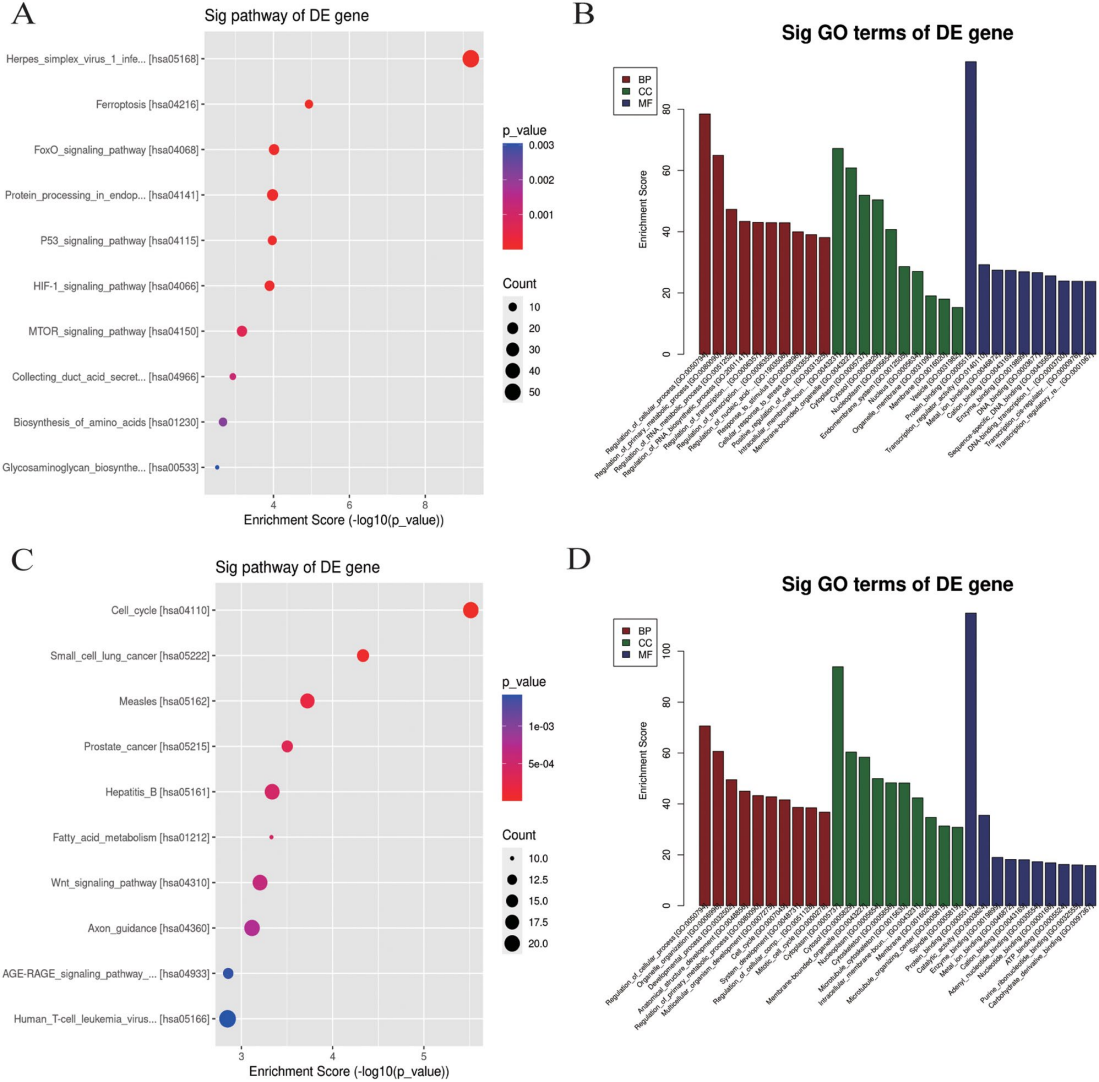
GO and KEGG analysis of DEmRNA host genes. **A**. KEGG enrichment analysis for host genes of these upregulated mRNAs. **B**. GO annotation for host genes of these upregulated mRNAs under the theme of BP, CC and MF. **C**. KEGG enrichment analysis for host genes of these downregulated mRNAs. **D**. GO annotation for host genes of these downregulated mRNAs under the The BP, CC and MF categories

Significant variations were found in the analysis of mRNA for GO and KEGG enrichment pathways. BP, CC, and MF were included in the GO analysis. The findings demonstrate that some mRNAs participate in crucial biological processes, such as controlling the cell cycle, mediating the HIF1a signal transduction pathway, and digesting proteins in the endoplasmic reticulum (Fig. 4). A KEGG enrichment analysis revealed that mRNAs with significant variations were crucial in controlling the cell cycle and influencing the signaling pathways for HIF1a, mTOR, WNT, and other signaling molecules.

### Construction of the ceRNA network

At present, the function of lncRNAs and circRNAs is not clear, and the prediction of their function mainly depends on mRNA. We queried miRNAs abnormally expressed in the placentas of PE and IUGR through the database. The target DEcircRNAs, DElncRNAs and DEmRNAs were predicted by miRNA. A circRNA-mRNA map and lncRNA-mRNA map were constructed, and a coexpression network map was further constructed.

**Fig. 5:**
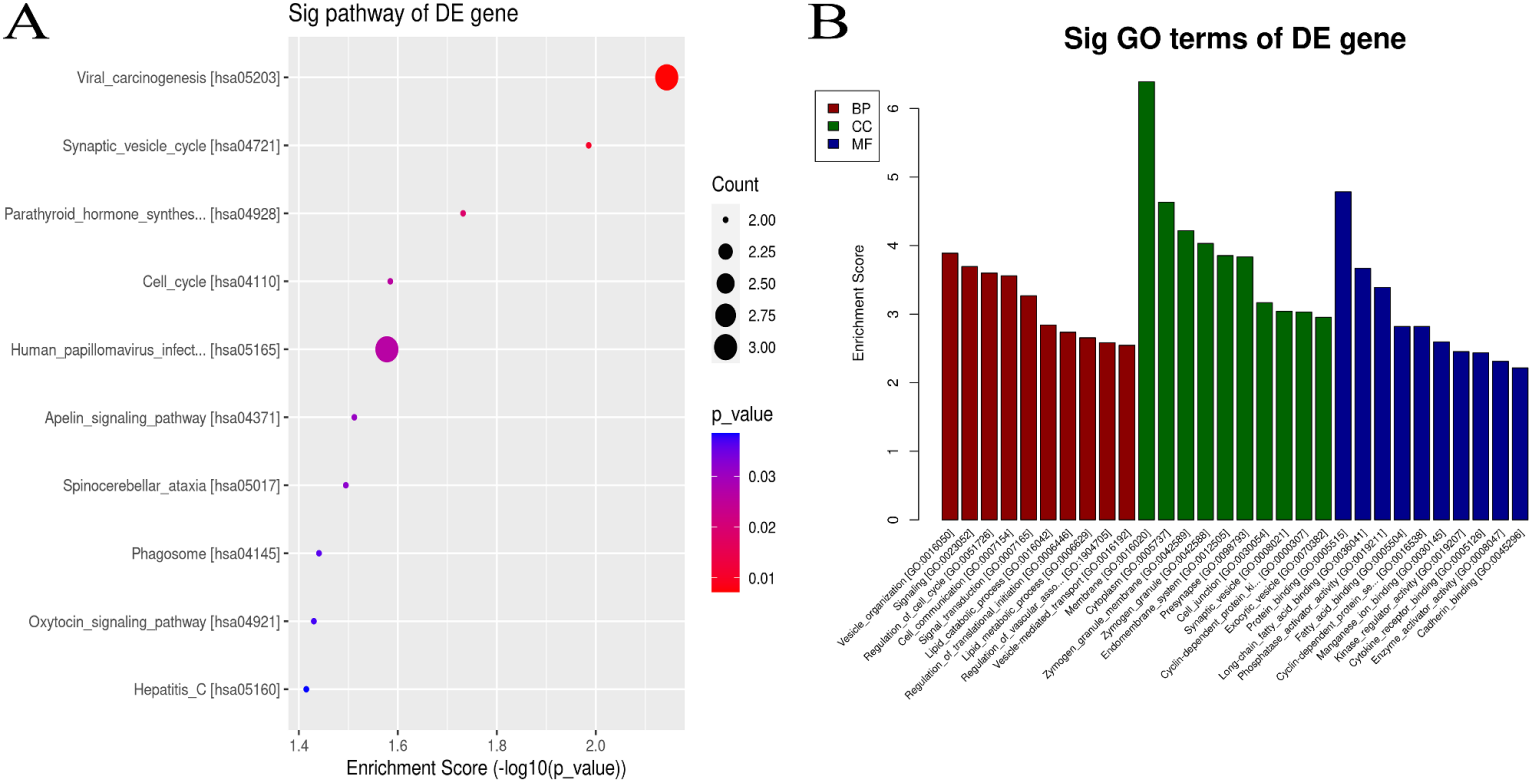
GO and KEGG analysis of DEcircRNA host genes. **A**. KEGG enrichment analysis for host genes of these DEcircRNAs. **B**. GO annotation for host genes of these DEcircRNAs under the theme of BP, CC and MF

As a sponge of miRNA, circRNA can regulate the function of miRNA, predict the target miRNA genes of circRNA with significant differences, and use these target genes for GO enrichment analysis, from BP, CC, MF three parts (Fig. 5). GO enrichment analysis of BP showed that these target genes are associated with MAPK, apelin, and oxytocin transduction. The GO analysis of CC showed that the genes were mainly located in the cell membrane and cytoplasm. Similarly, the KEGG pathway dot plot reveals the intermolecular interactions of gene-related pathways. The method with the highest enrichment fraction is shown.

**Fig. 6:**
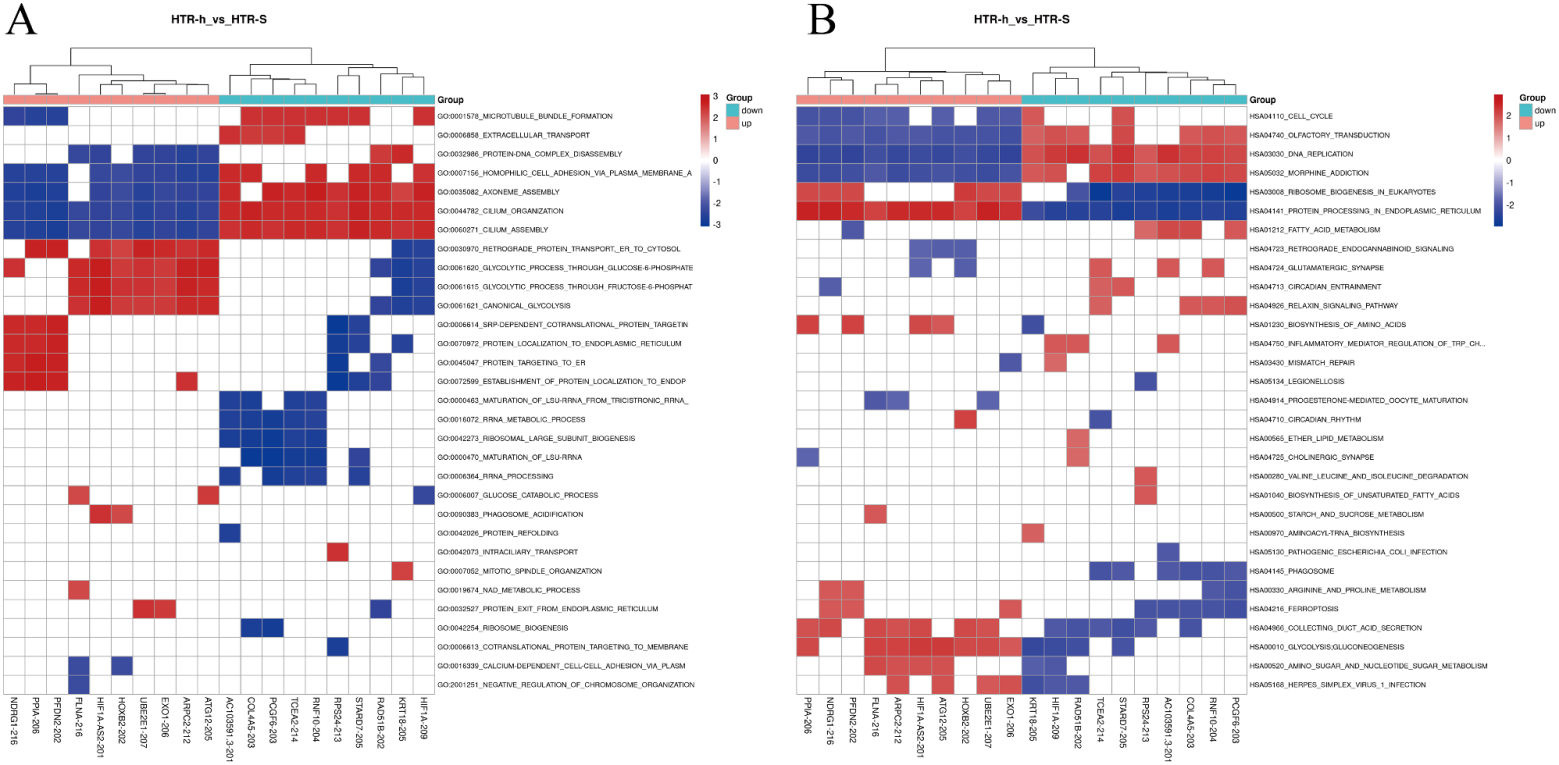
GO and KEGG analysis of DElncRNA host genes. **A**. GO annotation for host genes of these DElncRNAs under the theme of BP. **B**. KEGG enrichment analysis for host genes of these DElncRNAs.

Similarly, lncRNAs were analyzed, and GSEA showed biological function in anoxic HTR-8/SVneo cells (Fig. 6). The richest GO terms in the three categories were GO:0007156, GO:0035082, GO:0044782, and GO:0060271, which are associated with cell migration and invasion.

KEGG was mainly associated with cell cycle regulation, protein formation and processing, and cell proliferation.

## Discussion

miRNAs are thought to be a major factor in sickness caused by hypoxia, according to earlier research [23–25]. . Major hypoxia-induced miRNAs such as miR-210 and let-7 control angiogenesis, cell proliferation, the DNA damage response, and mitochondrial metabolism. Inhibiting cell proliferation, invasion, and angiogenesis, studies have shown that miR-210 is downregulated in preeclampsia and fetal placenta with intrauterine growth restriction [26–28]. Preeclampsia and intrauterine growth restriction are two maternal disorders caused by placental hypoxic stress, and the dysregulation of circRNAs plays a significant role in their pathogenesis. Recent research has revealed that dysregulated lncRNAs can be used as predictors and can play a significant role in maternal and baby disorders caused by hypoxia. The coexpression of lncRNAs and circRNAs as well as the ceRNA network are not fully understood, although there have been numerous pertinent studies [29–31].

To build the ceRNA network in this study, RNAseq was utilized to assess the expression profiles of lncRNAs, circRNAs, and miRNAs in human chorionic trophoblast cells under normal oxygen and hypoxic circumstances. A total of 2179 lncRNAs, 2263 mRNAs, and 41 circRNAs were differentially expressed between the study groups, according to RNA-seq analyses. According to KEGG pathway analysis, most genes were connected to the MAPK, mTOR, and HIF-1a signaling pathways, among others. These genes might be crucial for cell growth, invasion, and cycle processes.

The results of this study also showed that the expression levels of HOMX1 SLC7A5, HIF1a-AS2, SERPINE1 and ANGPTL4 were decreased in hypoxic cells, while the expression levels of AC103591.3, HIST1H2BM, HIST1H2BB, HIST1H1D and HIF1a were increased in hypoxic cells. These RNAs belong to the HIF1a signaling network. Vascular tone is related to HOMX1, and vascular regeneration is regulated by SERPINE1 [32]. Through the mTOR signaling pathway, SLC7A5 controls the production of amino acids [33]. Previous research has shown that the lncRNA HIF1a-AS2 improperly controls and regulates the expression of ANGPTL4 in PE, mediating involvement in invasion, apoptosis, and proliferation [20]. The results of this investigation demonstrated that the expression of HIF1a-AS2 and ANGPTL4 in hypoxia-induced HTR-8/SVneo cells was consistent.

**Fig. 7:**
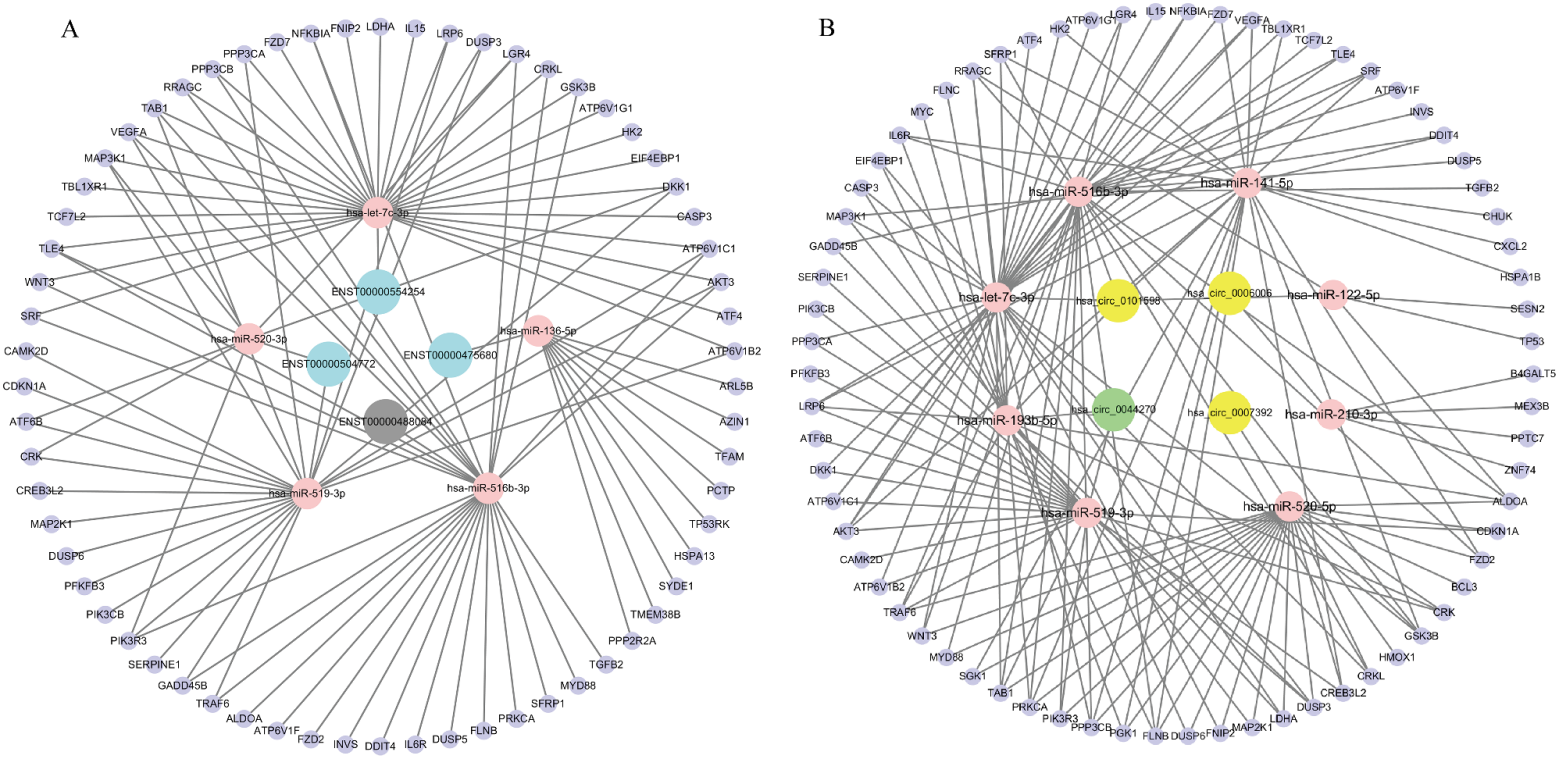
**A**. LncRNA-miRNA-mRNA network containing the high score interactions. The blue circles represent upregulated lncRNAs, the grey circles represent downregulated lncRNAs, and the pink circles represent miRNAs, and the purple circles represent mRNAs. **B**. CircRNA-miRNA- mRNA network containing the high score interactions. The yellow circle represents upregulated circRNAs, the green circles represent downregulated circRNAs, and the pink circles represent miRNAs, and the purple circles represent mRNAs

We chose a number of miRNAs that were misexpressed in the placentas of PE and IUGR pregnancies, predicted their regulatory relationships with their target genes, and then screened them (Fig. 7). The results showed 74 lncRNA-miRNA-mRNA interactions, of which 3 were upregulated, 1 was downregulated, 5 miRNAs, and 65 mRNAs. There were 82 circRNA-miRNA‒mRNA interactions; 8 miRNAs, 71 mRNAs, and 3 circRNAs were upregulated, and 1 was downregulated. Further screening of differentially expressed circRNAs, lncRNAs, and mRNAs controlled by the same miRNA was carried out in accordance with lncRNA-miRNA-mRNA and circRNA-miRNA-mRNA networks.

Recent research has demonstrated that lncRNAs can function as microRNA (miRNA) sponges to control posttranscriptional gene expression. For instance, in preeclampsia, the lncRNA DANCR can function as a competitive endogenous RNA to control trophoblast migration and invasion via miR-214-5p [34]. By targeting miR-330-5p in preeclampsia, lncRNA SNHG14 is involved in trophoblast proliferation, migration, invasion, and epithelial-mesenchymal transition [35]. As a rival RNA to miR-218-5p (ceRNA), lncRNA SNHG16 controls trophoblast invasion by specifically targeting LASP1 [36]. To better clarify the potential roles of lncRNAs in PE and IUGR, we created a lncRNA-miRNA-mRNA network.

Coexpressed lncRNA genes were investigated for functional pathway enrichment. We discovered that more targeted mRNAs were engaged in Mammalian target of rapamycin (mTOR), MAPK, TNF, WNT and other signaling pathways, controlling the cell cycle, proliferation, invasion, and apoptosis, which is consistent with the findings of the majority of research [37–39]. mTOR signaling pathway plays an important role in the placenta, participating in the invasion and migration of trophoblast cells, transport of nutrients and oxygen, and other important processes [40]. Wnt signaling pathway is involved in the pathophysiological process of severe PE by regulating the proliferation and invasion of trophoblast [41].These findings contribute to our understanding of the molecular processes by which these lncRNAs function.

According to reports, circRNAs play a role in the regulation of transcription and posttranscriptional gene expression [25]. They serve as miRNA sponges and are abundant in useful miRNA binding sites [42]. For instance, circ_0015382 controls the miR-149-5p/TFPI2 axis to control trophoblastic proliferation, migration, invasion, and epithelial-mesenchymal transformation (EMT) [43]. Through the upregulation of HOXD10 in trophoblast cells via miR-139-5p activity, circ_0077109 reduced the invasion and angiogenesis of trophoblast cells and increased apoptosis [44]. By controlling the miR-144/e-cadherin axis, circ_0085296 also prevents trophoblast cells from proliferating, invading, and migrating [45]. In this study, GO and KEGG analyses were used to examine defective circRNAs in anoxic culture HTR-8/SVneo cells. It was discovered that the pathways associated with cell proliferation, invasion, and the cell cycle, including the MAPK pathway associated with the cell cycle [46], the Toll-like pathway associated with the vesicle cycle [47], the PI3K-Akt signaling pathway associated with the apelin pathway [48, 49], the AMPK signaling pathway, and other pathways, may be crucial in the pathogenesis of diseases associated with hypoxia [50]. 

**Fig. 8:**
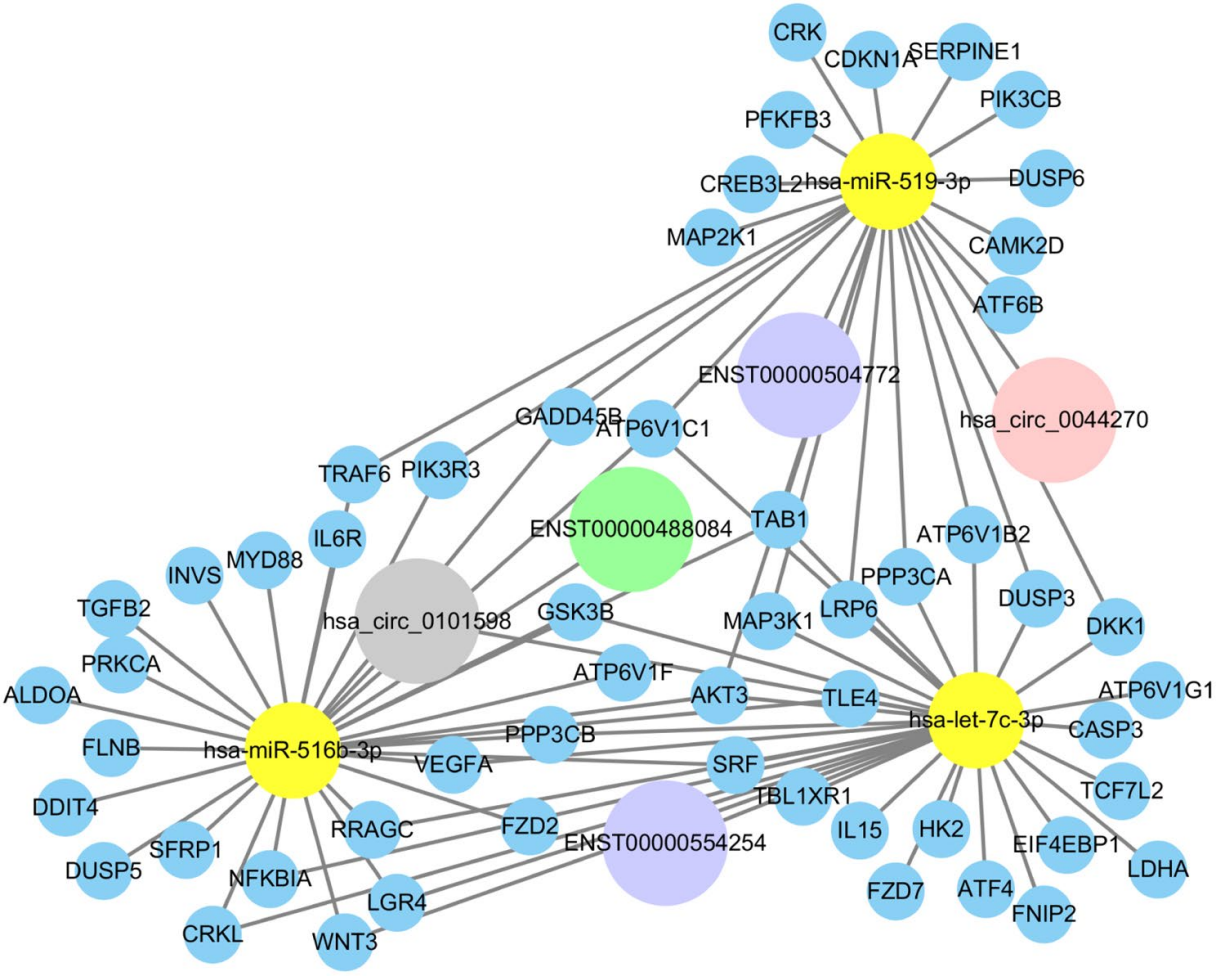
ceRNA network in hypoxic HTR-8/SVneo cells. The ceRNA network was based on lncRNA-miRNA, circRNA-miRNA and mRNA-miRNA interactions. In this network, lncRNA or circRNA expression is linked to mRNAs via miRNAs. The grey circle represents upregulated circRNAs, the dark pink circle represents downregulated circRNAs, the purple circle represents upregulated lncRNAs, the green circle represents downregulated lncRNAs, the yellow circles represent miRNAs, and the blue circles represent mRNAs

We further developed networks of coexpression between circRNAs and lncRNAs(Fig. 8). The results found that HIF1a-AS3 was upregulated in hypoxia-induced trophoblasts. Recent studies have implicated HIF1a-AS3 in regulating the ovarian cancer progression by binding to YBX1 [51]. However, except for HIF1a-AS3, other noncoding RNA with abnormal expressions in ceRNA have not been studied, and its biological functional effects on cells and the role of target gene axis still need to be further verified. Although no separate studies of these differential non-coding RNA exist, they were discovered to jointly induce let-7c and miRNA-516b, which primarily targeted the regulation of proliferation, invasion, and EVT-related mRNA and were found to be abnormally expressed in the placenta and serum of preeclampsia and fetal intrauterine growth restriction. This finding was in line with pertinent studies. In addition, the target genes of pe and IUGR-associated miRNAs are enriched in several important biological processes, including placental development, embryonic accessory morphogenesis, cell growth degree regulation, and vascular development [52–55].

Since the invention of RNA-seq, it has become clear that noncoding RNAs are crucial to the onset and development of illnesses. Although we have constructed a ceRNA regulatory network, there are still some limitations. Firstly, we conducted RNA-level differential analysis on the cells, but this analysis cannot guarantee the inclusion of all genes. Secondly, the ceRNA network we constructed is relatively limited. Further improvement is needed for the complete construction of the ceRNA network. Thirdly, Further experiments are needed to verify the functional impact of gene expression alterations.The profiles of these dysregulated lncRNAs and circRNAs can assist in discovering potential clinical markers and contribute to the knowledge of the pathogenesis and development of PE and IUGR, even though the findings of this work still need further experimental validation.

## Data Availability

The datasets and materials can be found at https://www.ncbi.nlm.nih.gov/geo/query/acc.cgi?acc=GSE248957.
